# Novel *Asaia bogorensis* Signal Sequences for *Plasmodium* Inhibition in *Anopheles stephensi*

**DOI:** 10.3389/fmicb.2021.633667

**Published:** 2021-02-16

**Authors:** Christina Grogan, Marissa Bennett, Shannon Moore, David Lampe

**Affiliations:** Department of Biological Sciences, Bayer School of Natural and Environmental Sciences, Duquesne University, Pittsburgh, PA, United States

**Keywords:** malaria, secretion, paratransgenesis, *Anopheles*, *Plasmodium*, *Asaia*

## Abstract

Mosquitoes vector many pathogens that cause human disease, such as malaria that is caused by parasites in the genus *Plasmodium*. Current strategies to control vector-transmitted diseases are hindered by mosquito and pathogen resistance, so research has turned to altering the microbiota of the vectors. In this strategy, called *paratransgenesis*, symbiotic bacteria are genetically modified to affect the mosquito’s phenotype by engineering them to deliver antiplasmodial effector molecules into the midgut to kill parasites. One paratransgenesis candidate is *Asaia bogorensis*, a Gram-negative, rod-shaped bacterium colonizing the midgut, ovaries, and salivary glands of *Anopheles* sp. mosquitoes. However, common secretion signals from *E. coli* and closely related species do not function in *Asaia*. Here, we report evaluation of 20 native *Asaia* N-terminal signal sequences predicted from bioinformatics for their ability to mediate increased levels of antiplasmodial effector molecules directed to the periplasm and ultimately outside the cell. We tested the hypothesis that by increasing the amount of antiplasmodials released from the cell we would also increase parasite killing power. We scanned the *Asaia bogorensis* SF2.1 genome to identify signal sequences from extra-cytoplasmic proteins and fused these to the reporter protein alkaline phosphatase. Six signals resulted in significant levels of protein released from the *Asaia* bacterium. Three signals were successfully used to drive the release of the antimicrobial peptide, scorpine. Further testing in mosquitoes demonstrated that these three *Asaia* strains were able to suppress the number of oocysts formed after a blood meal containing *P. berghei* to a significantly greater degree than wild-type *Asaia*, although prevalence was not decreased beyond levels obtained with a previously isolated siderophore receptor signal sequence. We interpret these results to indicate that there is a maximum level of suppression that can be achieved when the effectors are constitutively driven due to stress on the symbionts. This suggests that simply increasing the amount of antiplasmodial effector molecules in the midgut is insufficient to create superior paratransgenic bacterial strains and that symbiont fitness must be considered as well.

## Introduction

In 2019 there were an estimated 229 million cases and 409,000 deaths caused by human malaria (World Health Organization, 2020). This vector-borne disease continues to burden many countries in sub-Saharan Africa and India (World Health Organization, 2020). Malaria in humans is caused by several parasitic protist species belonging to the genus *Plasmodium* and vectored by *Anopheles* sp. mosquitoes (Wang and Jacobs-Lorena, 2013). Major control strategies include the use of insecticides to manage vector populations, insecticide treated bed nets to manage biting behavior, and antimalarial drugs to target the parasites in infected humans (Birkholtz et al., 2012; Benelli, 2015). Despite a decline in the number of malaria cases and deaths in recent years, the effectiveness of these measures has begun to decrease. Parasites have evolved resistance to common drug treatments, especially to artemisinin-based and partner drugs (Haldar et al., 2018). Mosquitoes, too, have evolved resistance to insecticides and adapted biting behaviors to avoid physical barriers (Sokhna et al., 2013). Clearly, additional strategies are needed in order to combat this deadly disease.

*Plasmodium* has a complex life cycle that requires an *Anopheles* mosquito vector and a human host. Newly proposed strategies target mosquitoes through population size reduction and alteration. One approach is to genetically modify the mosquito genome to create transgenic mosquitoes for release that reduce population size through gene drive mechanisms. Studies have shown success at engineering these mosquitoes to introduce genes that cause sterility or death in natural populations (Kitzmiller, 1972; Alphey, 2014; Adelman and Tu, 2016). Others have altered the genome to make the mosquitoes resistant to infection from the *Plasmodium* parasite (Wang and Jacobs-Lorena, 2013). However, these may prove difficult for field applications since a large number of *Anopheles* mosquito species can vector the pathogen, with some living in reproductively isolated populations (Favia et al., 1997; Raghavendra et al., 2011).

*Paratransgenesis* is another promising strategy for malaria control. Mosquitoes are hosts to a variety of microbial communities, and these symbiotic microorganisms can be used to affect the mosquito phenotype, specifically by engineering them to express antiplasmodials to kill the parasites in the mosquito. Strains of *Wolbachia pipientis* have been used for field applications within *Aedes aegypti* mosquitoes to inhibit the spread of dengue viruses (Moreira et al., 2009; Frentiu et al., 2014). While successful, these studies also highlight problems that can arise when using non-native microbes, such a deleterious impact on population dynamics or transmission issues due to the native symbionts in mosquito species that *Wolbachia* does not naturally inhabit (Hughes et al., 2014; Telschow et al., 2017). Researchers have explored multiple symbiotic microbes in mosquitoes for both vector control and pathogen transmission control. A densonucleosis virus found in *Anopheles* sp. was engineered to express green fluorescent protein in the mosquito host as proof of concept that viruses can be used for paratransgenesis (Ren et al., 2008). Two fungal species, *Beauveria bassiana* and *Metarhizium anisopliae* not only naturally infect and spread through mosquito populations, but have been shown to innately cause progressive mosquito death (Scholte et al., 2005; Bukhari et al., 2011). Furthermore, engineering the *Metarhizium anisopliae* fungi to express antimalarials showed a significant reduction in *Plasmodium* sporozoite counts within the mosquito host (Fang et al., 2011). However, roadblocks exist for these organisms since mosquito-specific viruses are host-specific and fungal survival in the midgut environment is low. The midgut provides the greatest bottleneck for the number of parasites, resulting in just 0–5 oocysts in field-caught mosquitoes even though thousands of parasites are typically ingested in the blood meal (Sinden, 1999; Shahabuddin and Costero, 2001; Sinden and Billingsley, 2001). This illustrates the need to use microbes that are native to the mosquito midgut for paratransgenesis since they are already adapted to surviving in the midgut conditions and would likely have little to no impact on midgut microbiota.

Several symbiotic bacterial species isolated from mosquitoes have been explored as potential paratransgenesis candidates for malaria. *Pantoea agglomerans* was engineered to secrete different antiplasmodial proteins and was able to inhibit *Plasmodium falciparum* development within the *Anopheles gambiae* mosquito host up 98% (Wang et al., 2012). While these results were promising, *P. agglomeran*s has no drive mechanism to spread throughout mosquito populations. Another candidate, *Serratia marcescens AS1*, is able to propagate through mosquito populations and can significantly reduce *Plasmodium falciparum* development when engineered to secrete antiplasmodials (Wang et al., 2017). However, strains of *Serratia marcescens* are major opportunistic pathogens in humans, causing many nosocomial infections every year (Grimont and Grimont, 1978; Mahlen, 2011; Samonis et al., 2011; Khanna et al., 2013; Sridhar et al., 2015; Fernández et al., 2020). An ideal paratransgenesis candidate would not only possess the ability to spread from one mosquito to another but would also lack human pathogenicity. *Asaia bogorensis* SF2.1 is a promising candidate that colonizes the ovaries, testes, salivary glands, and the midgut of the mosquito, and spreads both horizontally and vertically through mosquito populations (Favia et al., 2007; Damiani et al., 2010; Mancini et al., 2016). *Asaia* was first identified in the flowers of the orchid tree (*Bauhinia purpurea*) and of plumbago (*Plumbago auriculata*), and has even been found associated as spoilers of still natural product drinks and organic product enhanced packaged waters (Yamada et al., 2000; Horsáková et al., 2009). This bacterium colonizes a number of arthropods, especially those that feed on the nectar of plants, including *Anopheles stephensi*, *An. gambiae, An. maculipennis, Aedes aegypti*, *Ae. albopictus*, *Culex pipiens*, *Scaphoideus titanus*, and *Sogatella furcifera*, most of which can vector human diseases (Favia et al., 2007; Crotti et al., 2009; Chouaia et al., 2010; Ricci et al., 2012; De Freece et al., 2014; Li et al., 2020). Importantly, bacteria in the genus *Asaia* apparently cause very few human infections. Only a handful of rare cases have been reported, occurring in either severely immunocompromised patients or when it was directly injected into the bloodstream (Snyder et al., 2004; Tuuminen et al., 2006, 2007; Alauzet et al., 2010; Juretschko et al., 2010; Epis et al., 2012; Carretto et al., 2016).

A major challenge in developing paratransgenic strains of bacteria is the release of antiplasmodial peptides and proteins outside of the cell. In bacteria, many proteins reach the extracellular milieu via different one- or two-step secretion pathways (Christie, 2019). The two-step pathways rely on N-terminal signal peptides that direct proteins to the general secretory (Sec) or twin-arginine translocation (TAT) export apparatus in the inner membrane (IM) (Christie, 2019). Importantly, signal peptides can be predicted from a bacterial genome sequence based on their conserved features such as length, hydropathy profiles, and cleavage sites (Nielsen et al., 1997; Bagos et al., 2010; Petersen et al., 2011). We reasoned that increasing the level of antiplasmodial peptides released would lead to more efficient antiplasmodial bacterial strains. We report here the evaluation of twenty different *Asaia* Sec or TAT signal peptides for their ability to mediate what is likely to be non-specific, heterologous release from the periplasm and improve the ability of *Asaia* to act as a paratransgenesis platform. Increasing the amount of antiplasmodials released from the cell likely leads to greater suppression of parasites, but can also compromise the fitness of the resultant bacterial strains; therefore, careful strain construction should be considered.

## Materials and Methods

### Media and Antibiotics

For plasmid cloning, *E. coli* Top10F’ cells were cultured using standard Luria Bertani (LB) broth [1% tryptone, 0.5% NaCl, 0.5% yeast extract (w/v)] and LB agar (LB broth with 15 g/L agar). Media was supplemented with 30 μg/mL kanamycin. All *Asaia* strains were cultured in mannitol broth [0.5% yeast extract, 0.3% peptone, 2.5% mannitol (w/v)] or mannitol agar (mannitol broth with 15 g/L agar), both adjusted to a pH of 6.5 before sterilization. Davis minimal media broth [0.7% dipotassium phosphate, 0.2% monopotassium phosphate, 0.05% sodium citrate, 0.01% magnesium sulfate, 0.1% ammonium sulfate (w/v)] was used for cell collection and Davis minimal media agar (minimal broth with 15 g/L agar) supplemented with 0.5% (w/v) arabinose solution was used for colonization assessments. Media was supplemented with 120 μg/mL kanamycin for plasmid selection and also 100 μg/mL ampicillin for colonization assessments. Both liquid and solid media cultures for all strains were grown at 30°C, with agitation for liquid cultures. All bacterial strains and plasmids used in this study are described in Table 1.

**TABLE 1:** Strains and plasmids used in this study.

Strains	Characteristics		References
*E. coli* Top10F’	F′{lacIq, Tn10(TetR)} mcrA Δ(mrr-hsdRMS-mcrBC) Φ80lacZΔM15 ΔlacX74 recA1 araD139 Δ(ara leu) 7697 galU galK rpsL (StrR) endA1 nupG		Durfee et al. (2008)
*Asaia* SF2.1	Wild type strain isolated from *Anopheles* mosquitoes		Favia et al. (2007)

**Plasmids**	**Characteristics**	**Stable in *Asaia*?**	**References**

pNB92	Kan^*R*^, pBBR origin, P_*nptII*_ promoter, ‘*phoA* insert and MCS for secretion signal fusion construction	Yes	Bongio and Lampe (2015)
pNB95 (Sider)	pNB92 with siderophore receptor gene cloned	Yes	Bongio and Lampe (2015)
pNB97 (Siders)	pNB95 with siderophore receptor-scorpine-PhoA effector construct	Yes	Bongio and Lampe (2015)
pNB141	pNB92 with MYC tag-PhoA construct	Yes	Bongio (2015)
pDCP^1^	pNB92 with dipeptidyl carboxypeptidase II signal	Yes	This study
pGGTP	pNB92 with gamma-glutamyltranspeptidase signal	Yes	This study
pHyp1	pNB92 with hypothetical protein 1 signal	Yes	This study
pHyp2	pNB92 with hypothetical protein 2 signal	Yes	This study
pCG6 (TonB)	pNB92 with TonB dependent receptor protein 1 signal	Yes	Shane et al. (2018)
pTonB2	pNB92 with TonB dependent receptor protein 2 signal	No	This study
pHyp3	pNB92 with hypothetical protein 3 signal	No	This study
pAChan	pNB92 with ammonium channel/transporter	Yes	This study
pPIsom	pNB92 with peptidyl-prolyl *cis–trans* isomerase signal	Yes	This study
pHyp4	pNB92 with hypothetical protein 4 signal	Yes	This study
pHyp5	pNB92 with hypothetical protein 5 signal	No	This study
pALys	pNB92 with alginate lyase signal	Yes	This study
pHyp6	pNB92 with hypothetical protein 6 signal	Yes	This study
pTonB3	pNB92 with TonB dependent receptor protein 3 signal	Yes	This study
pHyp8	pNB92 with hypothetical protein 8 signal	Yes	This study
pPerox	pNB92 with peroxiredoxin signal	No	This study
pHyp9	pNB92 with hypothetical protein 9 signal	No	This study
pMXKDX	pNB92 with pentapeptide MXKDX repeat protein signal	No	This study
pABCTrans	pNB92 with ABC-type phosphate transport signal	Yes	This study
pCopB	pNB92 with copper resistance protein CopB signal	No	This study
pHyp1s	Hypothetical protein 1 signal-scorpine-(GGGGS)_3_- PhoA effector construct	Yes	This study
pTonBs	TonB dependent receptor 1 signal-scorpine-(GGGGS)_3_- PhoA effector construct	Yes	This study
pHyp4s	Hypothetical protein 4 signal-scorpine-(GGGGS)_3_- PhoA effector construct	Yes	This study

*Transgenic *Asaia* strains are referred to as the plasmid names. ^1^Plasmid sequences for plasmids constructed for this study were deposited into GenBank under accession numbers MW132102-MW132123.*

### Mosquito and Parasite Maintenance

*Anopheles stephensi* (a gift from the Johns Hopkins Malaria Research Institute) were maintained on 10% (w/v) sucrose solution at 29°C and 70% humidity with a 12 h day:12 h night light cycle. Larvae were reared at 29°C in pans and fed on crushed Tetramin Tropical Tablets for Bottom Feeders. Pupae were collected by hand and allowed to emerge as adults in 0.03 m^3^ screened cages. *Plasmodium berghei* strain ANKA2.34 was maintained by passage through 7- to 8-week-old outbred female ND4 Swiss Webster mice (Charles River Laboratory) using standard procedures (Sinden et al., 1996). This study was carried out in strict accordance with the recommendations in the Guide for the Care and Use of Laboratory Animals of the National Institutes of Health and Duquesne University IACUC protocol #1810-09. All surgery was performed using anesthesia as outlined below, and all efforts were made to minimize suffering.

### Genome Prediction of *Asaia* Extra-Cytoplasmic Proteins

An *Asaia bogorensis* SF2.1 genome sequence was annotated by the NCBI Annotation Pipeline version 2.0^1^ and a total of 3,005 protein-coding genes were identified (Shane et al., 2014). SignalP4.1 signal peptide prediction software was used to analyze the identified protein coding sequences (Nielsen et al., 1997). The top 20 predicted proteins are listed in Supplementary Table 1 (Bongio, 2015).

### Plasmid Construction

**FIGURE 1:**
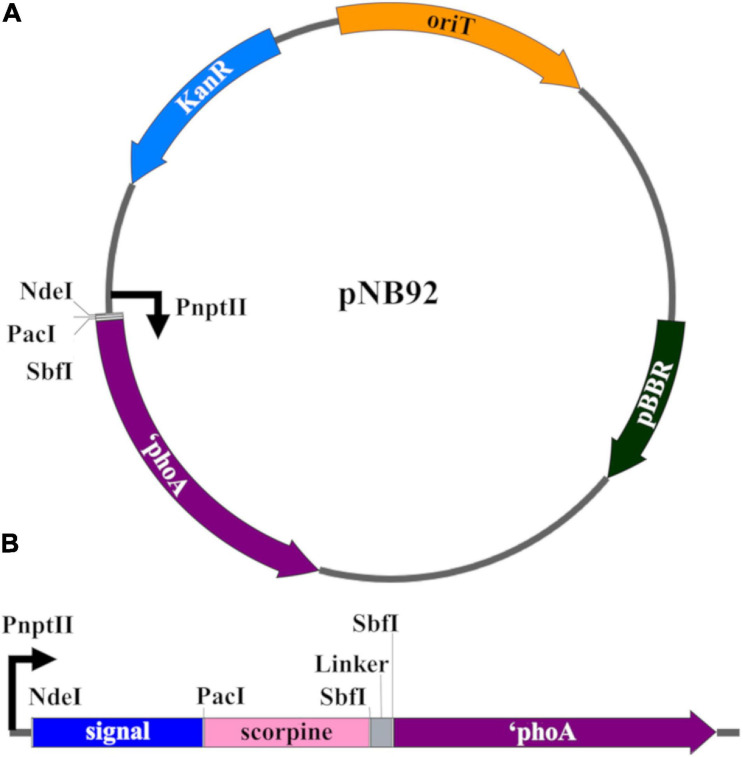
Genetic constructs for the creation of transgenic *Asaia* strains. **(A)** pNB92 vector backbone for secretion signal insertion (Bongio and Lampe, 2015). The first 150 nucleotides of predicted signal sequence were inserted between the *Nde*I and *Pac*I restriction sites for fusion with the reporter *E. coli* PhoA protein lacking its native signal sequence (phoA). pBBR, origin of replication; KanR, kanamycin resistance; oriT, origin of transfer; PnptII, promoter. **(B)** Construct for antiplasmodial effector strains. The ORF encoding the antimicrobial scorpine effector as well as a (GGGGS)_3_ flexible linker were inserted between the *Pac*I and *Sbf*I restrictions sites for fusion between the signal sequences and ‘*phoA* coding sequence.

All plasmid construction and propagation was performed in *E. coli* Top10F’ (Invitrogen). The pNB92 plasmid was used for construction of all *Asaia* sp. signal sequence vectors (Figure 1A) (Bongio and Lampe, 2015). This plasmid uses the pBBR broad-host range origin of replication, a constitutively active neomycin phosphotransferase promoter (P_*nptII*_), and the neomycin phosphotransferase II gene (nptII) conferring kanamycin resistance. A multiple cloning site after the P_*nptII*_ promoter allowed for in frame gene fusions with the *E. coli phoA* gene (without its native secretion signal = ‘*phoA*) to function as a reporter for protein localization (Manoil et al., 1990). Supplementary Table 2 lists the top 20 predicted exported proteins and their signal peptides that were identified from the *Asaia* SF2.1 genome (Bongio, 2015). Gblock (IDT) synthetic dsDNA fragments were designed conservatively, using the first 150 nucleotides of each protein and included *Nde*I and *Pac*I restriction digestion sites before and after these sequences, respectively (Supplementary Table 1). The pNB92 vector and each gblock were fully digested with *Nde*I and *Pac*I restriction enzymes and recovered from gel electrophoresis after size confirmation with the Gel/PCR Fragment Extraction Kit (IBI Scientific). Each gblock was individually assembled into the digested pNB92 vector using an optimized temperature-cycle ligation procedure and electroporated into *E. coli* Top10F’ electrocompetent cells (Lund et al., 1996). Clones were verified by PCR using the primer set in Supplementary Table 1, visualized through gel electrophoresis, and sequence verified. Plasmids were electroporated into *Asaia* SF2.1 electrocompetent cells, plated on selective media, and verified through PCR using the same primer set.

Plasmids expressing the antiplasmodial effector scorpine were constructed by digesting the plasmid pNB97 (Siders) and the constructs created above with the *Pac*I and *Sbf*I restriction enzymes (Conde et al., 2000; Bongio and Lampe, 2015). The scorpine gene fragment from pNB97 and the vector backbone from the signal sequence constructs were purified and assembled together using standard ligation procedures. A (Gly-Gly-Gly-Gly-Ser)_3_ flexible linker was also inserted between the scorpine and *‘phoA* gene fragments to promote proper folding of the fusion protein (Figure 1B). This double-stranded DNA fragment was achieved by annealing single-stranded complementary oligonucleotides together following the IDT standard protocol, which were designed with *Sbf*I restriction sites on both ends for ligation insertion (Supplementary Table 1). Briefly, the 500 μm oligonucleotide stock solutions were diluted to a final concentration of 100 μm in deionized water. The oligonucleotides were mixed in equal molar amounts, along with 2 μl of 10× Duplex Buffer [0.98% potassium acetate, 0.72% HEPES (w/v), pH 7.5] and deionized water to bring the final volume to 20 μl. The mix was heated to 94°C for 2 min and allowed to gradually cool. The mix was diluted 1:1000 in deionized water and 3 μl was used for ligation reactions. The products were transformed via electroporation into *E. coli* Top10F’ cells, PCR and sequence verified, and electroporated into *Asaia* SF2.1 and plated on selective media.

### Alkaline Phosphatase Reporter Screen

Passage of alkaline phosphatase (PhoA) across the inner membrane (IM) in *Asaia* was detected using 5-bromo-4-chloro-3′-indolyl phosphate (BCIP) supplemented media as previously described (Bongio and Lampe, 2015). Briefly, clones expressing the PhoA reporter constructs were plated on selective mannitol agar supplemented with 25 μg/mL BCIP and 30 μg/mL Na_2_HPO_4_. Since wild-type *Asaia* demonstrates a low-level of natural phosphatase activity, sodium phosphate was included as an inhibitor to reduce the number of false positives. Colonies were assessed for any color change after 72 h of growth at 30°C.

### ELISA

For the detection of PhoA in the cell culture fractions, the *Asaia* strains expressing the PhoA constructs were grown to an OD_600_ of 1.0. One mL of each culture was centrifuged at 2000 × *g* (5000 RPM) for 5 min. The supernatant was removed and saved while the cell pellet was washed three times and resuspended in 1 mL Tris-buffered saline (TBS) [0.605% Tris-Cl, 0.876% NaCl, pH 7.5 (w/v)]. A second cell pellet from the same culture and isolated at the same time was resuspended in 1 mL of a 20% B-Per-TBS (v/v) solution (Thermo-Fisher Scientific, #78243) and vortexed for 2 min to lyse the cells. Two hundred microliters of the supernatant, whole cell, and cell lysate fractions were bound in wells of a NUNC-Immuno Maxisorp 96-well plate (VWR, cat. # 62409-024) overnight at 4°C. Plates were washed three times with TBS, and then blocked by adding 200 μl of 2% BSA-TBS (TBS with 2% (w/v) fraction V BSA) and incubating for 2 h at room temperature. The plate was again washed three times with TBS. Then, 100 μl of a 1:3000 dilution of rabbit polyclonal anti-PhoA-HRP antibody (GeneTex, #GTX27319) in 2% BSA-TBS was added to each well and incubated for 1 h at room temperature. The plate was washed eight times with 0.1% Tween20 (v/v)-TBS (TBS-T) for 2 min per wash. To visualize the protein, 50 μl of 1-Step Ultra TMB-ELISA (Thermo-Fisher Scientific, #34028) was added and the reaction allowed to proceed for 10–20 min at room temperature. To stop the reaction, 50 μl of 2 M H_2_SO_4_ was added, and the absorption at 450 nm was measured using a SpectraMax i3x plate reader (Molecular Devices), using the absorption at 655 nm for reference. These assays were performed five separate times and the results analyzed by one-way ANOVA with Dunnett’s correction.

### Western Analysis

*Asaia* strains expressing the PhoA constructs were grown overnight. Cultures were streaked on mannitol plates and grown at 30°C for 48 h. Colonies were collected by flooding the plates with 1 mL of minimal media, gently scraping the cells from the plate, and collecting the liquid into centrifuge tubes. These samples were then centrifuged at 13,800 × *g* (12,000 RPM) for 5 min. The supernatants were collected and placed on ice while the cell pellet was resuspended in 1 mL of 20% B-Per-TBS. The total protein concentration for each of the pelleted samples was analyzed through a Bradford assay (Thermo-Fisher Scientific, #23236) after accounting for any excess protein concentration from the mannitol medium. A dilution factor was established for each pellet sample to achieve a concentration of 1200 μg/ml for the pellet. The same dilution factor was then applied to the supernatant. In this way, the supernatant fractions were scaled relative to each other based on the total protein content of the cell pellet from which they were derived. Seventy-five microliters of each adjusted supernatant was added to 25 μl of 3× Laemmli buffer and the samples were boiled for 8 min.

Fifteen microliters of each sample and 8 μl of Precision Plus Protein Kaleidoscope ladder (Bio-Rad cat. #161-0375) were loaded onto on a 10% Mini-PROTEAN TGX Precast gel (Bio-Rad) and separated at 200 V for 35 min. Proteins were then transferred onto a PVDF membrane in a Bio-Rad transfer apparatus using Tris-glycine transfer buffer [0.303% (w/v) Tris, 1.127% (w/v) glycine, 10% methanol (v/v)] at 100 V for 1 h. The membrane was dried overnight, then was stained for total protein as a loading control using Revert 700 total protein stain (LI-COR cat. #926-11010) following the manufacturer instructions and imaged using an Odyssey FC dual mode imaging system (LI-COR) using the 700 nm infrared fluorescent detection channel for 2 min. The membrane was then rinsed in water and then blocked with 50% v/v Odyssey blocking buffer in TBS [50% (v/v) fraction V LI-COR Odyssey Buffer with TBS] for 1 h at room temperature with agitation and rinsed three times in deionized water for 5 min. The membrane was incubated in the primary antibody solution containing a 1:5,000 mouse monoclonal anti-PhoA antibody (Millipore, Temecula, CA, United States, MAB1012) diluted in 50% v/v Odyssey blocking buffer in TBS-T overnight at 4°C with agitation. The next day, the membrane was washed three times for 10 min with TBS-T. It was incubated for 1 h at room temperature with agitation in the secondary antibody solution containing a 1:20,000 IRDye^®^ 800CW goat monoclonal anti-mouse antibody (LI-COR, cat. # 925-32210) diluted in 50% v/v Odyssey blocking buffer in TBS-T with 0.01% w/v SDS. Two washes for 10 min with TBS-T was followed by one wash for 10 min with TBS. The membrane was visualized on an Odyssey FC dual mode imaging system (LI-COR) using the 800 nm infrared fluorescent detection channel for 2 min. Image Studio Software 5.0 (LI-COR) was used for blot visualization and band quantification. In order to normalize the amount of protein in each lane of the western blot, the amount of protein in each entire lane was quantified, the lane with the most protein determined, and each lane of the blot scaled to the highest amount. This scaling factor was then applied to each of the bands quantified using the anti-PhoA antibody.

### Fitness Assessments of *Asaia* Strains

Two methods were used to assess the fitness of *Asaia* strains. First, the maximum growth rate of each strain was measured following the procedure outlined in Shane et al. (2018). Each *Asaia* strain was inoculated at 0.1 OD_600_ in 200 μl of a 96 well plate. The OD_600_ was analyzed over 24 h at 15 min intervals using a SpectraMax i3x (Molecular Devices). SoftMax Pro 7 software (Molecular Devices) was used to create growth curves of collated replicates of each strain until they reached stationary phase. Growth curves were further analyzed using the package growthrates59 (Petzoldt, 2017) in RStudio to find the maximum growth rate of each strain of *Asaia*. Data was visualized in RStudio using boxplot.

A mosquito colonization experiment was also used to assess the fitness of the strains. Each *Asaia* strain was fed to female *An. stephensi* mosquitoes at a 0.1 OD_600_ dilution in a sugar meal. After 36 h, mosquito midguts were dissected and homogenized using a tissue grinder. Fifteen midguts for each strain were pooled and diluted in 1000 μl of TBS. These samples were again diluted 10-fold in TBS and 100 μl of each dilution was plated on kanamycin and ampicillin supplemented minimal media with arabinose. CFUs for each strain were counted and compared to the total number of CFUs collected across test groups. Data was visualized in RStudio using boxplot.

### *Plasmodium berghei* Parasite Inhibition

The ability of the antiplasmodial *Asaia* strains to inhibit *Plasmodium berghei* development in mosquitoes was evaluated according to Shane et al. (2018). Adult female ND4 Swiss Webster mice were infected with *P. berghei* ANKA2.34 and parasites were allowed to develop in the mice until parasitemia level reached 4–10%. At this point the mice were sacrificed and blood was collected via cardiac puncture. The infected blood was diluted with RPMI media (Gibco) to 2% parasitemia, then 200 μl (5 × 10^7^ parasites) was injected intraperitoneally into an uninfected mouse. At the time of this transfer, each *Asaia* scorpine strain to be tested was diluted to 0.1 OD_600_ in the sugar meal and fed to 20–25 female *An. stephensi* mosquitoes in individual cups with screen lids. Thirty-six h post-infection each test group of mosquitoes was blood-fed on the infected mouse for 6 min each. The ability of the parasite to undergo exflagellation was also tested at this time using 6 μl ookinete media [1 L RPMI media supplemented with 0.2% sodium bicarbonate, 0.005% hypoxanthine, 0.00025% xanthurenic acid (w/v)] mixed with 10% (v/v) fetal bovine serum, 2 μl of 1 mg ml^–1^ of heparin in sterile phosphate buffer (PBS) [0.8% NaCl, 0.02% KCl, 0.144% Na_2_HPO_4_, 0.024% KH_2_PO_4_, pH 7.2 (w/v)], and 2 μl of blood collected from a tail prick of the mouse. At least two exflagellation events occurred for each malarial trial. Exflagellation occurs when microgametes exit red blood cells after a female mosquito takes a *Plasmodium*-infected blood meal, and can be monitored by microscopy. The number of these events in the blood meal is a measure of how infectious it is to the mosquito.

Mosquitoes that did not take in a blood meal were removed, and parasites were allowed to develop in the rest of the mosquitoes for 14 days at 19°C in order to form oocysts. After 14 days, the mosquito midguts were dissected and stained with a 10-fold dilution of 1% (v/v/) mercurochrome stain (Sigma Aldrich Product# M7011) in PBS for 30 min. They were then left to destain for 5 min in sterile PBS. The midguts were analyzed at 200× magnification and the number of oocysts per midgut were counted for each test group. All steps in this process were performed blindly and ordered randomly. Data was visualized in RStudio using Bee Swarm.

### Statistics and Reproducibility

For all boxplots, the box bars are medians. The top and bottom of the boxes represent the first and third quartile of the data spread. The lower and upper bounds of the whiskers are the lowest datum still within 1.5× interquartile range (IQR) of the lower quartile, and the highest datum still within 1.5× IQR of the upper quartile, respectively. Significance for all tests was set to *P* < 0.05. Variance was estimated using standard error of the mean and is appropriately similar between test groups of each experiment. Significance of the mean was calculated using one-way ANOVA with Dunnett’s correction in RStudio appropriate for multiple comparisons to a single control with normal distribution unless otherwise noted.

In Figure 7, Suppression of *P. berghei* development by paratransgenic *Asaia* strains, the data are pooled from three individual experiments. The median value of oocysts per midgut for the pooled data from the three experiments was calculated by and compared between treatments using quantile regression in RStudio (Cade and Noon, 2003). Quantile regression is a non-parametric test that compares subsets of a data set individually and is useful for data showing unequal variation (Cade and Noon, 2003). The significance of the difference in *P. berghei* oocyst prevalence was evaluated using binomial χ^2^ tests with 1 degree of freedom. All colony and oocyst counts were done blindly regarding which strain was evaluated, and the strains were ordered randomly.

## Results

### *Asaia* Genome Prediction of Exported Proteins

Annotation of the *Asaia* SF2.1 genome identified 3,005 total protein-coding genes (Shane et al., 2014). SignalP4.1 signal peptide prediction software further identified 228 proteins which were predicted to be exported to the periplasm via the Sec or the Tat system (Bongio, 2015). Among the top 20 predictions were peptidyl-dipeptidase DCP and gamma-glutamyltranspeptidase secreted proteases, multiple TonB dependent receptor proteins and an ammonium transporter protein, a peptidyl-prolyl *cis–trans* isomerase folding chaperone, and the digestive enzyme alginate lyase (Supplementary Table 2). A siderophore receptor protein (Sider) that had been isolated previously in an *Asaia* genomic library screen using the reporter protein alkaline phosphatase (PhoA) was not identified from this genomic analysis (Bongio and Lampe, 2015). While these proteins are predicted to be exported past the inner membrane, some may only be localized to the periplasm or the outer membrane. We decided to focus our attention on the top 20 proteins with the highest scores.

### Alkaline Phosphatase Protein Localization Using *Asaia* Signal Sequences

The vector pNB92 was used for construction of reporter constructs using the signals from the top 20 putative secreted protein genes (Bongio and Lampe, 2015). This vector contains the gene coding the *E. coli* PhoA protein lacking its native signal peptide (Figure 1A). The first 150 nucleotides of each signal were inserted in front of the *‘phoA* gene, allowing for fusion of the predicted signal peptides to the PhoA reporter protein. These constructs were transformed into *Asaia* SF2.1 cells to create new strains that are referred to following the names of the plasmids they are carrying (Table 1). Thirteen of the *Asaia* transformants grew while seven did not, even after repeated attempts at transformation, indicating that the latter might be toxic when overexpressed in *Asaia* in these configurations (Table 1).

The successful clones were first screened for alkaline phosphatase activity by plating them on 5-bromo-4-chloro-3′-indolyl phosphate (BCIP) supplemented agar. BCIP can detect PhoA exported across the IM of Gram-negative cells, thus indicating that PhoA could be actively cleaving BCIP as a secreted protein, a membrane bound protein, or from within the periplasm (Brockman and Heppel, 1968). From this screen, nine strains showed a deep blue color change after 3 days of growth (Hyp1, Hyp2, TonB, AChan, PIsom, Hyp4, Hyp6, TonB3, and Hyp8), indicating cleavage of BCIP in the medium. The wild type *Asaia* SF2.1 colonies had no color change.

**FIGURE 2:**
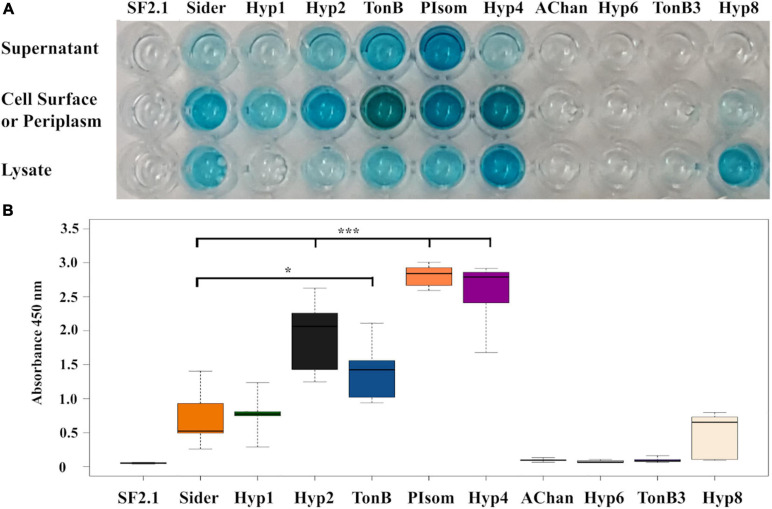
Alkaline phosphatase localization in transgenic *Asaia* strains using ELISA. **(A)** A representative ELISA that utilized an anti-PhoA-HRP antibody to detect the presence of the alkaline phosphatase protein in the supernatant, cell surface/periplasm, and the cell lysate fractions of PhoA-only *Asaia* strains. SF2.1, wild-type *Asaia*; Sider, previously identified siderophore receptor signal (Bongio and Lampe, 2015). **(B)** Quantification of supernatant fractions of the ELISA analysis. Relative levels (expressed as absorbance values measured at 450 nm) of substrate cleaved by the HRP-conjugate anti-PhoA antibody in the supernatant fraction across five separate trials. Statistical significance was determined using one-way ANOVA with Dunnett’s correction where significance is represented by *P < 0.05, **P < 0.01, and ***P < 0.001 with experimental replicates. SF2.1, wild-type *Asaia*; Sider, previously identified siderophore receptor signal (Bongio and Lampe, 2015).

For the clones that showed a color change, an ELISA was performed to determine if PhoA was being released beyond the outer membrane (Figure 2A). Cultures were separated into the supernatant, whole cell, and cell lysate fractions, and the protein of interest was detected using an anti-PhoA-HRP conjugated antibody. Of the nine clones identified in the BCIP screen, six strains (Hyp 1, Hyp2, TonB, PIsom, Hyp4, and Hy8) showed PhoA protein in the supernatant and/or the whole cell fractions. Four of these (Hyp2, TonB, PIsom, and Hyp4) showed significantly more PhoA protein in the supernatant when compared to the previously identified siderophore receptor (Bongio and Lampe, 2015) Sider signal peptide (Figure 2B; *P* ≤ 0.0209). The other three strains AChan, TonB3, and Hyp6 exhibited no detectable PhoA protein in any fraction.

**FIGURE 3:**
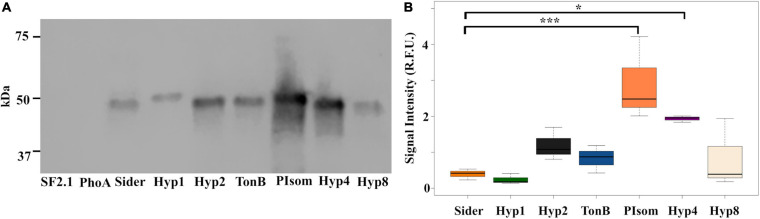
Abundance of alkaline phosphatase protein in the supernatants from *Asaia* using predicted N-terminal signal peptides. **(A)** Representative western blot of supernatants from transgenic *Asaia* strains that showed localization of the PhoA reporter protein to the supernatant in the ELISA. The PhoA protein was detected using a monoclonal anti-PhoA antibody. **(B)** PhoA protein abundance in the supernatant. Western blots for all strains were repeated three times, and the signal intensities for the PhoA band were quantified without using a correction for lane to lane variation in total protein as an internal control. Statistical significance was determined using one-way ANOVA with Dunnett’s correction where significance is represented by *P < 0.05, **P < 0.01, and ***P < 0.001 with experimental replicates. No protein was detected in the SF2.1 or the PhoA (with no signal sequence) lanes. For the other strains, only significant comparisons are shown. SF2.1, wild-type *Asaia*; PhoA, alkaline phosphatase with no signal sequence; Sider, previously identified siderophore receptor signal. The same analysis correcting for variations in total protein between lanes as an internal control is shown in Supplementary Figure 2.

In order to further quantify the amount of protein in the supernatant relative to that of Sider, a western blot analysis was carried out on the supernatant of the six strains that were positive for PhoA in the ELISA using the anti-PhoA antibody. No signal was seen in the lanes with the SF2.1 strain and the PhoA strain with no signal sequence (=PhoA), while the rest of the supernatants had a small protein band around 49.9 kDa, which is the predicted size of the full PhoA protein after signal cleavage (Figure 3A).

Quantification of this analysis introduces a methodological problem. Typical western analysis uses internal controls of some housekeeping protein(s) whose amounts do not vary under different conditions (Yosef et al., 2010; Wu et al., 2012). No such control is available for the supernatant fraction that we are analyzing here. Protein mass normalization between lanes can be achieved by using total protein stains as an alternative (e.g., Revert 700 Total Protein Stain, LI-COR). We performed that control, but in this analysis we do expect variations in the amount of protein in the supernatant, so the suitability of this particular control is suspect. In our analysis, supernatant amounts varied such that they reflected the total protein content of the cell pellets from which they were derived. We therefore quantified the amount of PhoA in the supernatant produced by each strain with and without total protein normalization (Figure 3B and Supplementary Figure 2). The overall pattern of the relative protein amounts without total protein stain normalization essentially reproduced the results of the ELISA assay albeit with some differences in significances. Quantification of the PhoA-reactive bands without normalization showed that both the PIsom and Hyp4 strains had significantly increased amount of PhoA protein in the supernatant (one-way ANOVA with Dunnett’s correction, *P* ≤ 0.0387) when compared to the Sider signal peptide, while Hyp2 and TonB were not significantly different although they were significantly different in the ELISA (*P* ≥ 0.4474) (Figure 3B). Quantification of the western analysis with total protein normalization did not reproduce the results of the ELISA and showed only Hyp4 as releasing significantly more protein than did Sider (one-way ANOVA with Dunnett’s correction, *P* = 0.00443) (Supplementary Figure 2).

### Fitness Assessments of Alkaline Phosphatase *Asaia* Strains

**FIGURE 4:**
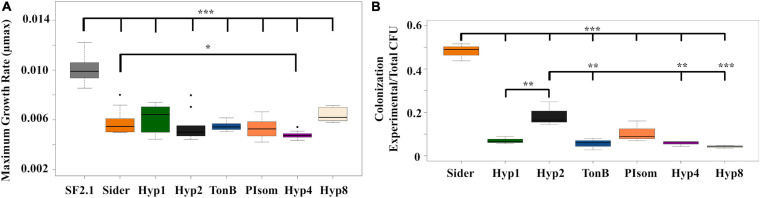
Fitness assessments of alkaline phosphatase *Asaia* strains. **(A)** Growth curves and maximum growth rates (μmax) were calculated from 10 individual isolates of each strain grown over log phase of the bacteria using the package growthrates59 (Petzoldt, 2017) in RStudio. Only Hyp4 showed a significantly lower μmax than the Sider strain. **(B)** Relative colonization of mosquito midguts by *Asaia* strains. *Asaia* strains were fed to mosquitoes and 15 midguts from each sample were pooled and plated on selective media. Transgenic CFUS were counted for each isolate and taken as a ratio of the total across all strains. The Sider strain had the highest rate of colonization, followed by the Hyp2 strain. Statistical significance for each experiment was determined using one-way ANOVA with Dunnett’s correction where significance is represented by *P < 0.05, **P < 0.01, and ***P < 0.001 with experimental replicates (**A**. *n* = 10, **B**. *n* = 3). Only significant comparisons are shown.

Two fitness assessments were carried out on the PhoA strains to determine if any were at a disadvantage when compared to the *Asaia* SF2.1 strain. First, the assessment of the maximum growth rates (μmax) of the strains were compared (Figure 4A). None of the transgenic strains grew as well as the *Asaia* SF2.1 wild-type strain (one-way ANOVA with Dunnett’s correction, *P* ≤ 0.001). When the newly constructed strains were compared to the Sider strain, only Hyp4 showed a significantly lower μmax (one-way ANOVA with Dunnett’s correction, *P* = 0.0423).

The relative ability of the strains to colonize the mosquito midgut was also assessed (Figure 4B). The transgenic strains were fed to mosquitoes, midguts were dissected and plated under conditions that selected for *Asaia* growth, and CFUs were counted across all strains. None of the new transgenic strains were able to colonize the midgut as well as the Sider strain (one-way ANOVA with Dunnett’s correction, *P* < 0.001). The Hyp2 strain colonized the midgut significantly better than four of the other new transgenic strains (one-way ANOVA with Dunnett’s correction, *P* ≤ 0.0469).

### Antiplasmodial Effector Release Using *Asaia* Signal Sequences

**FIGURE 5:**
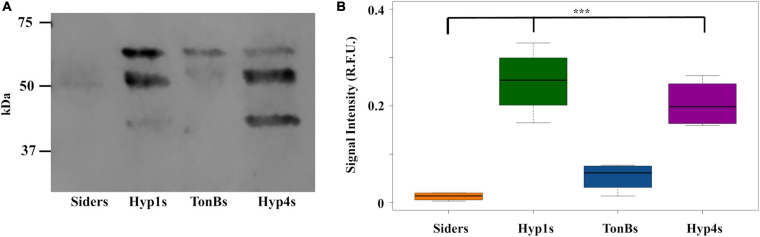
Antiplasmodial effector abundance in the supernatants from *Asaia*. **(A)** Representative western blot of supernatants from antiplasmodial *Asaia* strains. The PhoA segment of the fusion protein was detected using a monoclonal anti-PhoA antibody. Three bands were detected with the antibody. The larger band (57 kDa) corresponds to the size of the scorpine-PhoA protein, while we interpret the other two bands (51 and 47 kDa) as most likely corresponding to cleavage products. **(B)** Fusion protein abundance in the supernatant. Western blots for all strains were repeated four times, and the signal intensities for the bands were quantified without using a correction for lane to lane variation in total protein as an internal control and reported as a single value. Statistical significance was determined using one-way ANOVA with Dunnett’s correction where significance is represented by *P < 0.05, **P < 0.01, and ***P < 0.001 with experimental replicates. Only significant comparisons are shown. SF2.1, wild-type *Asaia*; Siders, scorpine antiplasmodial strain using the previously identified siderophore receptor signal. Similar analyses to this figure using a correction for variations in total protein in each lane and quantifying only the largest reactive band in each lane are shown in Supplementary Figures 4–6.

An ORF encoding the antimicrobial peptide scorpine (Conde et al., 2000; Carballar-Lejarazú et al., 2008) was introduced into the six vectors that showed PhoA localization in the supernatant in the ELISA and western blot assays. A flexible linker was inserted between the scorpine and *‘phoA* gene fragments to promote proper folding and the independent function of each part of the fusion protein (Figure 1B). Attempts to create antiplasmodial *Asaia* strains with the Hyp2, PIsom, and Hyp8 signals proved problematic, resulting in no colonies on the selective plates after multiple transformation attempts suggesting that these configurations were toxic to *Asaia*. Western blot analysis was carried out on the supernatant of the new antiplasmodial strains Hyp1s, TonBs, and Hyp4s as well as Siders, the antiplasmodial strain using the siderophore receptor signal (Table 1). The supernatants for Hyp1s, TonBs, and Hyp4s showed two or three prominent bands, the largest one corresponding to the predicted size of the intact scorpine-PhoA protein at 57 kDa (Figure 5A). The other two bands were at approximate sizes of 51 kDa and 47 kDa and are most likely the product of additional cleavage within the protein either within the periplasm or as the protein exits the cell (Figure 5A).

Quantification of the protein released into the supernatant was carried out for all strains. The protein normalization problem discussed earlier for the strains expressing only PhoA is relevant here as well. In addition, the fact that the scorpine-PhoA fusion appears to be proteolytically cleaved means that accurately assessing the amount of scorpine reaching the supernatant is problematic since our reporter tag becomes separated from scorpine.

In order to estimate the amount of scorpine leaving the cell, we performed two different kinds of measurements. In the first, we quantified all of the protein fragments that reacted to the anti-PhoA antibody and reported that as a single value for the antiplasmodial strains (Figure 5B and Supplementary Figure 4). This measurement assumes that the scorpine that was cleaved also left the cell and so the smaller forms of reactive PhoA are a proxy for the presence of scorpine. We also quantified only the largest band which corresponds to the scorpine-PhoA fusion and thus is a direct measure of scorpine presence outside the cell in that form (Supplementary Figures 5, 6). There are, thus, four separate ways to measure the amount of scorpine that has left the antiplasmodial *Asaia* strains depending on the assumptions used. Figure 5B shows data for one of these sets of assumptions, namely allowing the amount of protein in the supernatant to vary (but normalized to the amount of protein in the cell pellet) and quantifying all of the antibody reactive PhoA protein bands assuming that all of the scorpine originally translated as a fusion protein left the cell. Under these conditions, Hyp4s showed significantly more protein than Siders (one-way ANOVA with Dunnett’s correction, *P* < 0.001). Hyp1s also exhibited significantly more protein than the Siders strain (one-way ANOVA with Dunnett’s correction, *P* < 0.001), in contrast to the Hyp1 PhoA only strain result (Figure 3B). The TonBs strain also showed full protein in the supernatant fraction, but not to a significantly greater degree than Siders (one-way ANOVA with Dunnett’s correction, *P* = 0.465).

Analysis of the western results using the other three sets of assumptions is shown in Supplementary Figures 4–6. Under all sets of assumptions, Hyp1s released more scorpine than did Siders (one-way ANOVA with Dunnett’s correction, *P* ≤ 0.00681). TonB does as well when measured without total protein normalization quantifying only the largest reactive band (one-way ANOVA with Dunnett’s correction, *P* = 0.0456).

### Fitness Assessments of Paratransgenic *Asaia* Strains

**FIGURE 6:**
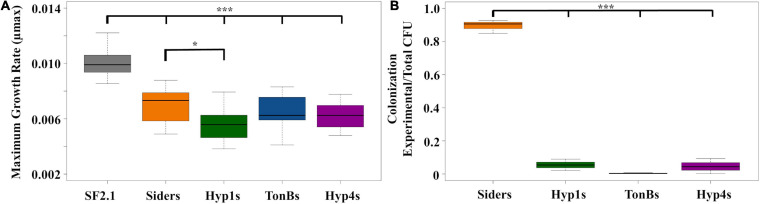
Fitness assessments of antiplasmodial *Asaia* strains. **(A)** Maximum growth rates (μmax) were calculated from 10 individual isolates of each strain grown over log phase of the bacteria using RStudio. Only Hyp1s showed a significantly lower μmax than the Siders strain. **(B)** Relative colonization of mosquito midguts by antiplasmodial *Asaia* strains. Antiplasmodial *Asaia* strains were fed to mosquitoes and 15 midguts from each sample were pooled and plated on selective media. Transgenic CFUS were counted for each isolate and taken as a ratio of the total across all strains. Siders showed the highest rate of colonization. Statistical significance for each experiment was determined using one-way ANOVA with Dunnett’s correction where significance is represented by *P < 0.05, **P < 0.01, and ***P < 0.001 with experimental replicates (**A**. *n* ≥ 10, **B**. *n* = 3). Only significant comparisons are shown.

Fitness assessments were carried out on the antiplasmodial strains to determine if any of the new strains showed a loss of fitness when compared to both Siders and the *Asaia* SF2.1 wild-type control. First, the maximum growth rates (μmax) of the strains were compared (Figure 6A). None of the antiplasmodial strains grew as well as the *Asaia* SF2.1 strain (one-way ANOVA with Dunnett’s correction, *P* ≤ 0.001). The Hyp1s strain showed a decrease in μmax compared to Siders while the other two antiplasmodial strains did not (one-way ANOVA with Dunnett’s correction, *P* < 0.0165).

In addition, the relative ability of the antiplasmodial strains to colonize the mosquito midgut was assessed (Figure 6B). The antiplasmodial strains were fed to mosquitoes, midguts were dissected and plated, and CFUs were counted across all strains. Once again, the Siders had the highest rate of colonization in the midgut with the other three strains showing a substantially lower rate (one-way ANOVA with Dunnett’s correction, *P* < 0.001).

### Activity of Paratransgenic *Asaia* Strains Against *Plasmodium berghei*

**FIGURE 7:**
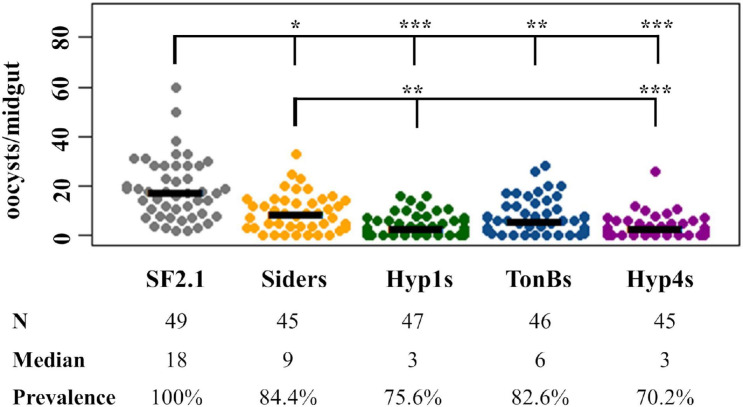
Suppression of *P. berghei* development by paratransgenic *Asaia* strains. In three separate trials, oocysts were counted in mosquitoes infected with *Asaia* strains that were fed on a *P. berghei* infected mouse. Each dot represents an individual midgut and the number of *P. berghei* oocysts it contained. Prevalence is the fraction of midguts with at least one oocyst. SF2.1 is the wild type *Asaia* strain (negative control) and Siders is the scorpine antiplasmodial strain using the previously identified siderophore receptor signal (positive control). All antiplasmodial strains significantly reduced the median number of oocysts (horizontal bars) compared to the wild-type strain (quantile regression, *P* < 0.001). The Hyp1s and the Hyp4s strains showed a significant reduction in the median number of oocysts (the bottom set of horizontal comparisons in the figure) compared to the Siders strain (quantile regression, *P* ≤ 0.00187). The prevalence of infection (the top set of horizontal comparisons in the figure) was also significantly different between the wild-type and all antiplasmodial strains (χ^2^, 1 df). There was no significant difference in prevalence of infection between the Siders strain and any of the new antiplasmodial strains (χ^2^, 1 df). *P*-values: *P < 0.05, **P < 0.01, ***P < 0.001.

The three antiplasmodial strains of *Asaia* developed here (Hyp1s, TonBs, and Hyp4s) were tested for their ability to prevent the development of *P. berghei* oocysts in *An. stephensi* female mosquito midguts, along with the Siders and SF2.1 strains as controls. The paratransgenic and wild-type strains were fed to female *An. stephensi* mosquitoes, which in turn fed on a *P. berghei* infected mouse. Mosquitoes that successfully blood-fed were dissected 14 days after the infective blood meal, and oocysts per midgut were counted (Figure 7). All of the paratransgenic strains significantly reduced the median number of oocysts when compared to the SF2.1 wild-type control *Asaia* strain (quantile regression, *P* ≤ 0.00029). Hyp1s and Hyp4s had a significantly greater median oocyst reduction compared to the Siders strain (quantile regression, *P* ≤ 0.00181), indicating that the increased levels of scorpine in the midgut had a stronger antiplasmodial effect in the mosquitoes. Another measure of antiplasmodial activity is prevalence. Prevalence is the fraction of mosquitoes of a population that have at least one oocyst, meaning that they may still be infective and can pass on the *Plasmodium* parasite. All of the antiplasmodial strains showed a significant reduction in prevalence of *P. berghei* infection when compared to SF2.1 (15.6–29.8%). However, there was no significant difference in prevalence between the Siders strain and the Hyp1s, TonBs, and Hyp4s antiplasmodial strains (χ^2^, *P* ≥ 0.1684).

## Discussion

Malaria continues to plague humans in tropical areas across the globe (World Health Organization, 2020). Current preventative strategies have led to the evolution of mosquito and parasite resistance, and new strategies are desperately needed (Liu, 2015; Haldar et al., 2018). Two proposed strategies focus on reducing the vectoral capacity of mosquitoes through either genetically altering the mosquito genome itself or through *paratransgenesis* whereby symbiotic microorganisms are genetically modified to affect the mosquito’s phenotype (Kitzmiller, 1972; Alphey, 2014; Adelman and Tu, 2016). Many barriers exist for the use of transgenic mosquitoes given that several species of mosquitoes vector malaria parasites, population genetic considerations like reproductive isolation, and allelic variation within gene drive target sites that may render them ineffective (The malERA Consultative Group on Vector Control, 2011; Wang and Jacobs-Lorena, 2013; Carballar-Lejarazú and James, 2017). Paratransgenesis may be more advantageous due to the ease of engineering the symbionts, ease of dispersal to natural populations, and lower fitness costs to the mosquito host itself (Wang et al., 2017; Bilgo et al., 2018). Many paratransgenesis candidates have been proposed, including bacterial symbionts such as *Pantoea agglomerans* and *Serratia marcescens AS1*; when engineered to express antiplasmodial effectors, these bacteria were able to significantly inhibit *Plasmodium* parasite infection while having little to no impact on the host mosquito mortality and fecundity compared to wild-type strains (Wang et al., 2012, 2017). Here, we looked to improve paratransgenesis using *Asaia bogorensis* SF2.1 by isolating novel signal peptides and examining their ability to deliver the antiplasmodial peptide, scorpine, outside the cell. *Asaia* is not naturally antiplasmodial so it must be genetically engineered to produce antiplasmodials.

Previous work investigated the use of signal peptides in *Asaia* SF2.1 to deliver antiplasmodial effector molecules within mosquito vectors. Common bacterial signal sequences such as *E. coli* OmpA and those from closely related species *Gluconobacter oxydans* and *Gluconacetobacter diazotrophicus* were originally tested in *Asaia*, but none mediated export of a reporter protein (Bisi unpublished; Bongio, 2015). A genetic library screen from *Asaia* SF2.1 yielded only one signal, a siderophore receptor signal (=Sider), that was successful in the release of antiplasmodial effectors (Bongio and Lampe, 2015). Driving the export of the antimicrobial scorpine with the identified siderophore signal led to significant *Plasmodium* oocyst inhibition, but only reduced the prevalence, or the fraction of mosquitoes of a population that have at least one oocyst, by 20% (Bongio and Lampe, 2015). Prevalence is important since a mosquito with even one oocyst may still be infective and can pass on the *Plasmodium* parasite. The *Asaia* genomic fusion containing the signal sequence is also very long, at over 500 amino acids in length, which could have an increased fitness cost to the bacterial strains that carry it. Therefore, we hypothesized that increasing the amount of toxin released from the cell using different N-terminal signal peptides would lead to significantly improved levels of paratransgenesis.

In this study, we isolated six new signals that mediated measurable release of the reporter protein, PhoA. When used to mediate the release of an antiplasmodial scorpine-PhoA fusion protein, the behavior of the new signals was unpredictable. For example, no transformation could be obtained with scorpine-PhoA fusions fused to either the Hyp2, PIsom, or Hyp8 signals, even though these constructs were stable in *E. coli*. Another signal, Hyp1, performed poorly when fused only with PhoA, while it was one of the better performers when fused to scorpine-PhoA. Based on these results, further use of the signals in *Asaia* to drive the release of other antiplasmodials will most likely have to be evaluated empirically.

Scorpine is a strong antimicrobial molecule that seems to share properties of both cecropin and defensin (Conde et al., 2000). Though the exact mechanism by which it interrupts *Plasmodium* and other microbial cells is unknown, its cationic properties are thought to contribute to binding to the negatively charged lipids on membranes to cause disruption and eventually cell lysis (Carballar-Lejarazú et al., 2008). It has also been shown to affect the fitness of *Asaia*, so constitutive expression using these constructs might be toxic for the cells and may need to be regulated (Shane et al., 2018). Other antiplasmodials that are specific to *Plasmodium* can also be considered for use to limit fitness costs to the bacterium, such as SM1 and EPIP, a *Plasmodium* enolase–plasminogen interaction peptide (Ghosh et al., 2001, 2011).

We relied on western analysis to determine the amount of scorpine released by strains constructed with the new signal peptides. Proteolytic cleavage, however, appeared to occur which separated the reporter PhoA from the antiplasmodial scorpine making relative concentrations of scorpine released difficult to quantify precisely. The known antiplasmodial behavior of scorpine and the mosquito midgut experiment performed here offer some insight on how to interpret these data, however. Purified scorpine is known to be highly active against *Plasmodium berghei* mosquito life stages (gametocytes and ookinetes) in a dose-dependent manner (Conde et al., 2000). Indeed, the ED_50_ of scorpine was 0.7 μM against ookinetes, the stage that actively invades midgut cells and leads to the formation of oocysts (Conde et al., 2000). Thus, more scorpine in the midgut is expected to correlate to greater parasite killing power. The mosquito experiments performed here identified two strains of *Asaia* (Hyp1s and Hyp4s) that significantly decreased the median number of oocysts per midgut beyond that produced by our previous strain using the signal from the siderophore receptor (Figure 7). These two strains also released significantly more scorpine when measured by the western analysis that assumed that all of forms of PhoA could be used as a proxy for the amount of scorpine released from the cell (Figure 5B). These data are not conclusive since they depend upon particular assumptions regarding western quantification, but they do suggest that increasing the amount of scorpine released from *Asaia* strains improves paratransgenesis.

Paratransgenesis in *Asaia* would likely be dramatically improved if we could achieve *bona fide* secretion in this species. Although signal peptides are relatively easy to predict from primary sequence data due to their N-terminal position and structure (Nielsen et al., 1997; Bagos et al., 2010; Petersen et al., 2011), identifying proteins that are actually secreted is much more difficult because there are no universal markers for secretion in mature bacterial proteins, although verified substrates secreted by particular bacterial secretion systems are known (Eichinger et al., 2016; An et al., 2017; Zeng and Zou, 2019). Newer *in silico* tools like Bastionhub are becoming available to predict secreted proteins (Wang et al., 2021) and these can be expected to aid the development of native secretion systems in the various bacterial species that are being developed for paratransgenesis, including *Asaia*.

In conclusion, we successfully identified and tested six new *Asaia* signal sequences that delivered heterologous protein outside the cell. These should prove useful to create new paratransgenic strains in the future, especially since paratransgenic strains for field release will necessarily need to produce more than one effector protein to decrease the chances of parasites evolving resistance to any single effector. Our simple hypothesis that increasing the amount of antiplasmodial proteins released from *Asaia* strains would lead to improved paratransgenesis was difficult to prove conclusively given that the scorpine-PhoA fusion protein underwent proteolytic cleavage separating the reporter from the antiplasmodial peptide. In addition, expression of heterologous proteins using these new signals generally decreased the fitness of the *Asaia* strains. Even so, this research brings *Asaia* one step closer for field-readiness and shows the attractiveness of paratransgenesis and the ease of its implementation, which can easily be combined with the other measures for both vector and parasite control.

## Data Availability Statement

The datasets presented in this study can be found in online repositories. The names of the repository/repositories and accession number(s) can be found below: https://www.ncbi.nlm.nih.gov/genbank/, MW132102–MW132123 and https://datadryad.org/stash, doi: 10.5061/dryad.tx95x69wj.

## Ethics Statement

The animal study was reviewed and approved by Duquesne University Institutional Animal Care and Use Committee.

## Author Contributions

CG designed the experiments, built the *Asaia* strains, tested them for protein localization and antiplasmodial effects, and wrote the manuscript. MB and SM performed the fitness experiments. DL designed the experiments and co-wrote the manuscript. All authors contributed to the article and approved the submitted version.

## Conflict of Interest

The authors declare that the research was conducted in the absence of any commercial or financial relationships that could be construed as a potential conflict of interest.

## Footnotes

1 https://www.ncbi.nlm.nih.gov/genome/annotation_prok/
