# Supplementary material for: Novel Asaia bogorensis Signal Sequences for Plasmodium Inhibition in Anopheles stephensi
Source: Front Microbiol. 2021 Feb 16;12:633667. doi: 10.3389/fmicb.2021.633667 (PMC7921796; doi:10.3389/fmicb.2021.633667)
Supplement: Supplementary file 1 [file Data_Sheet_1.pdf]

Supplementary Material

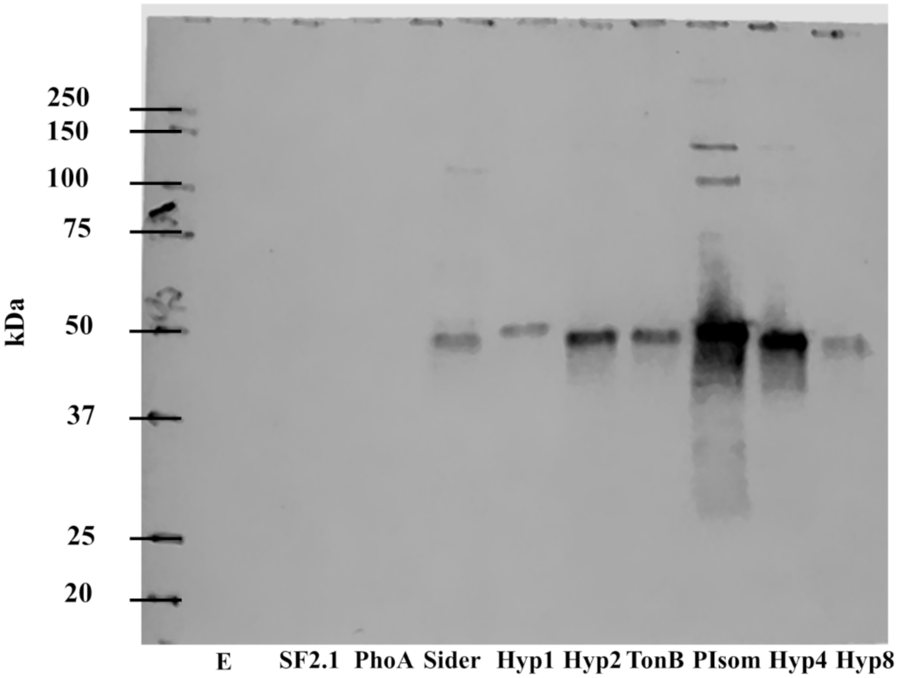

Figure S1: **Original uncropped western blot corresponding to Figure 3A in the manuscript, marked in the same manner.** E=empty lane. SF2.1=wild-type *Asaia*. PhoA=alkaline phosphatase with no signal sequence. Sider=previously identified siderophore receptor signal.

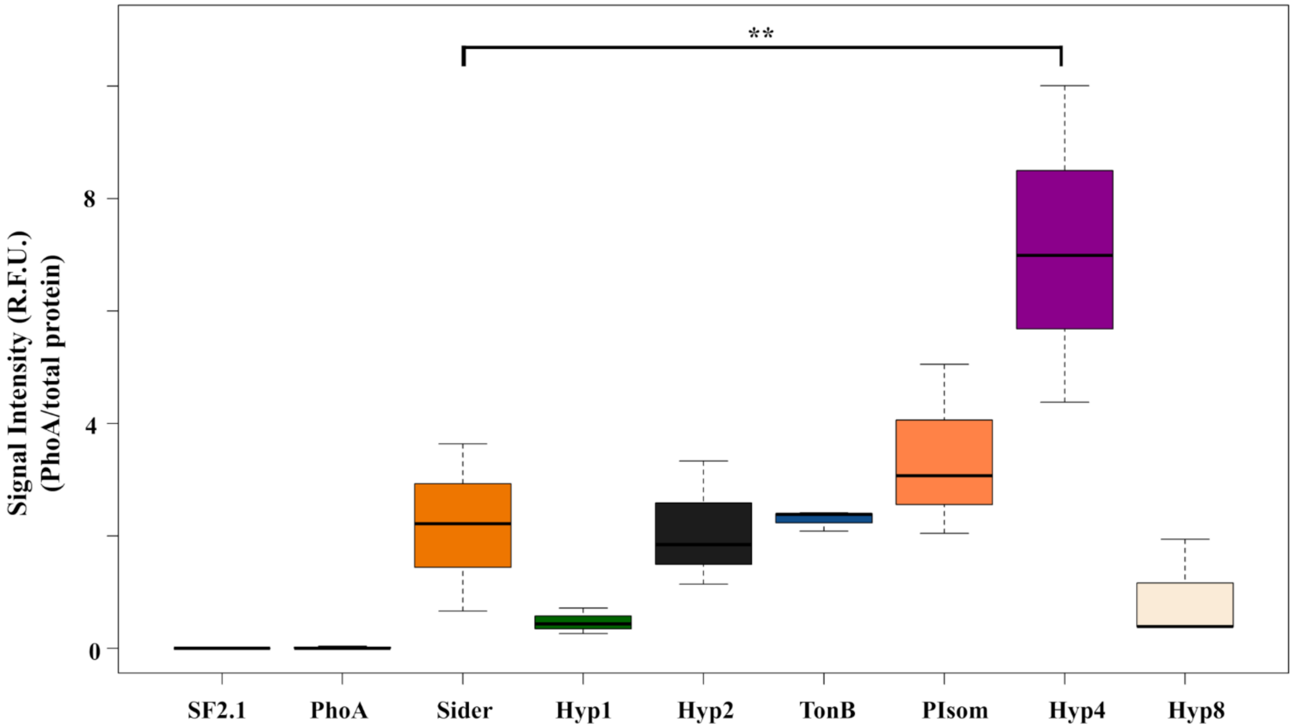

Figure S2: **PhoA protein abundance in the supernatant after total protein normalization**. Western blots for all strains were repeated three times, and the signal intensities for the PhoA bands were quantified with normalization to total protein as a correction for lane to lane variation. Statistical significance was determined using one way ANOVA with Dunnett's correction where significance is represented by *$P < 0.05$, **$P < 0.01$, and ***$P < 0.001$ with experimental replicates. No protein was detected in the SF2.1 or the PhoA (with no signal sequence) lanes. For the other strains, only significant comparisons are shown. SF2.1=wild-type *Asaia*. PhoA=alkaline phosphatase with no signal sequence. Sider=previously identified siderophore receptor signal.

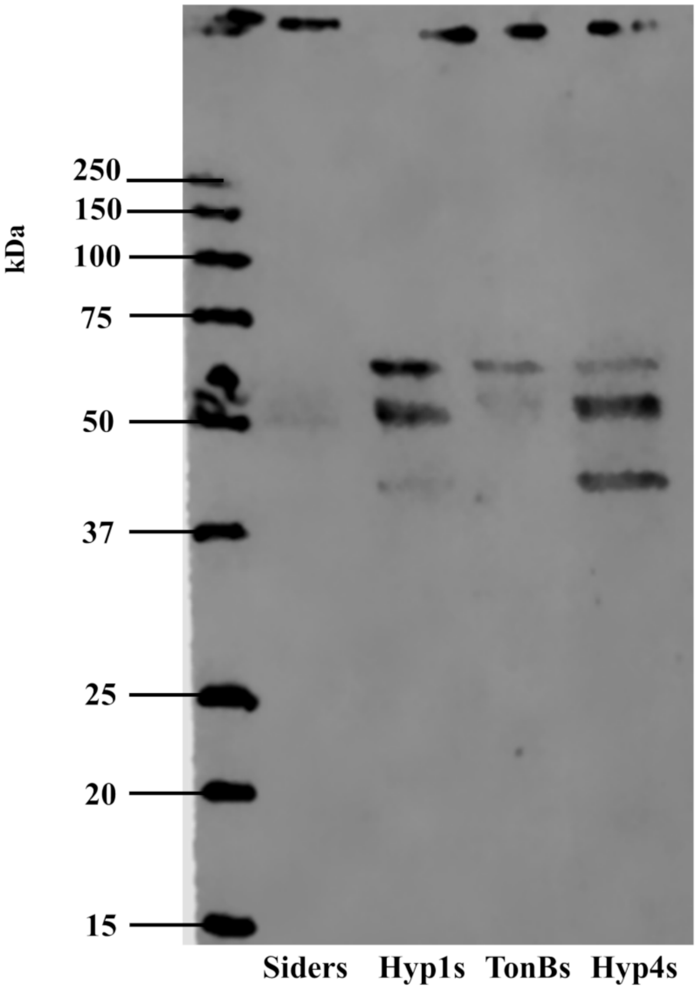

Figure S3: **Original uncropped western blot corresponding to Figure 5A in the manuscript, marked in the same manner.** Siders=scorpine antiplasmodial strain using the previously identified siderophore receptor signal.

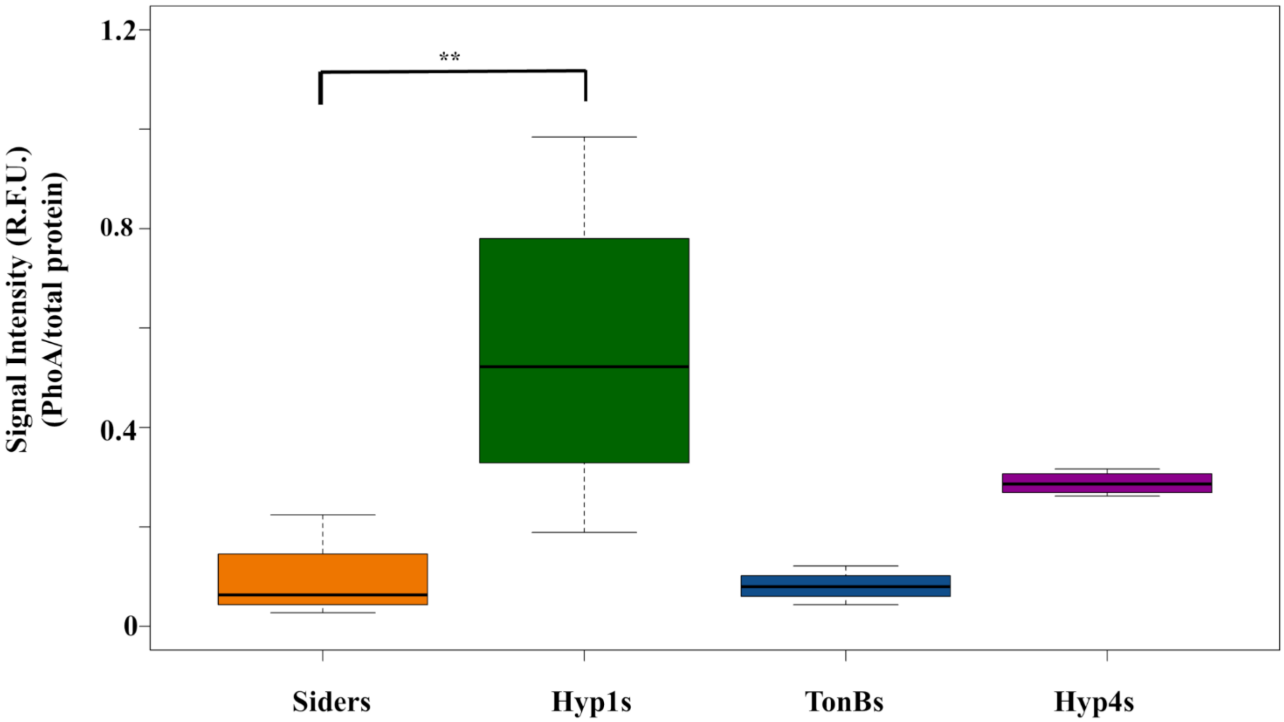

Figure S4: **Antiplasmodial effector abundance in the supernatants from *Asaia* after total protein normalization.** Western blots for all strains were repeated four times, and the signal intensities for all three bands were quantified with normalization to total protein as a correction for lane to lane variation and reported as a single value. Statistical significance was determined using one way ANOVA with Dunnett's correction where significance is represented by $*P < 0.05$, $**P < 0.01$, and $***P < 0.001$ with experimental replicates. Only significant comparisons are shown. Siders=scorpine antiplasmodial strain using the previously identified receptor signal. Similar analyses to this figure without a correction for variations in total protein in each lane and quantifying only the largest reactive band in each lane are shown in the manuscript Figure 5B and Supplemental Figures S5 and S6.

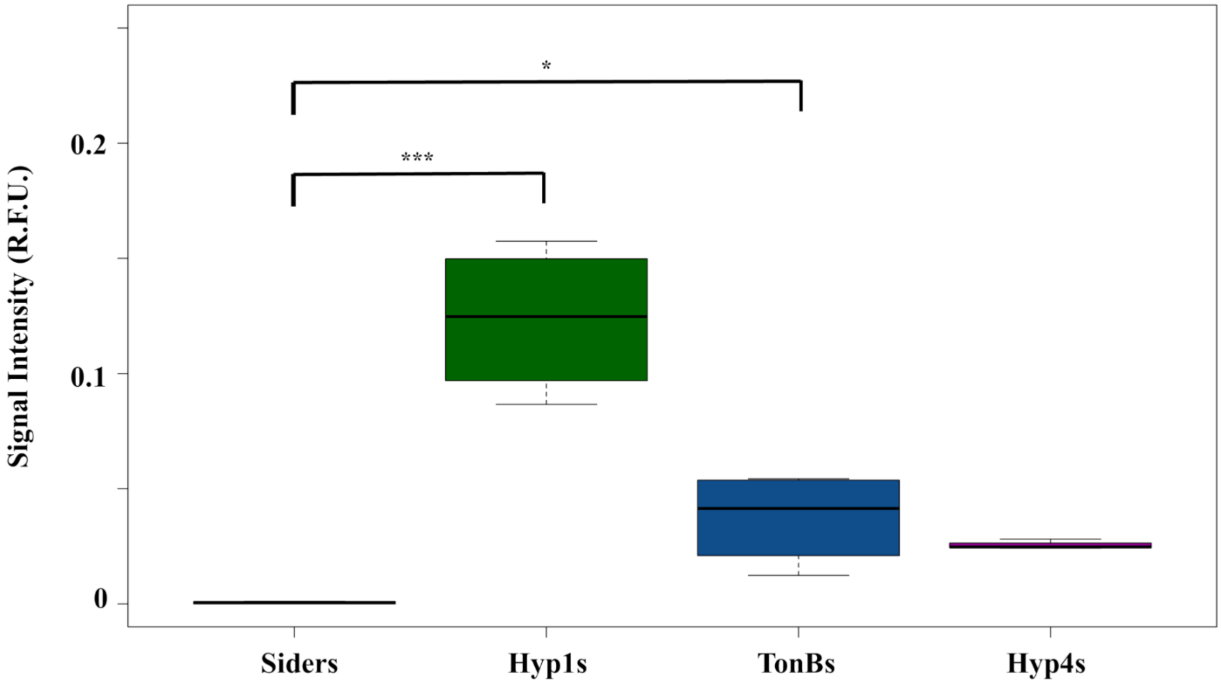

Figure S5: **Full-length antiplasmodial effector abundance in the supernatants from *Asaia*.** Western blots for all strains were repeated four times, and the signal intensity for only the full length scorpine-PhoA fusion band was quantified without using a correction for lane to lane variation. Statistical significance was determined using one way ANOVA with Dunnett's correction where significance is represented by *$P < 0.05$, **$P < 0.01$, and ***$P < 0.001$ with experimental replicates. Only significant comparisons are shown. Siders=scorpine antiplasmodial strain using the previously identified siderophore receptor signal. Similar analyses to this figure using a correction for variations in total protein in each lane and quantifying all three reactive bands in each lane are shown in the manuscript Figure 5B and Supplemental Figures S4 and S6.

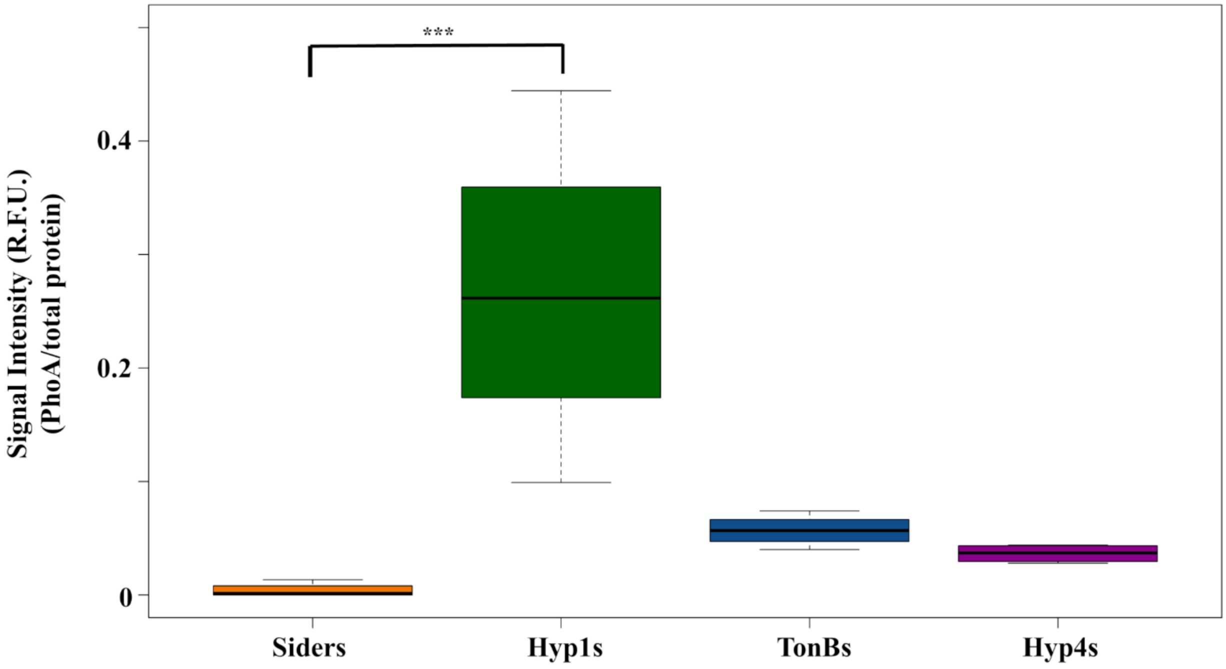

Figure S6: **Full-length antiplasmodial effector abundance in the supernatants from *Asaia* after total protein normalization.** Western blots for all strains were repeated four times, and the signal intensity for only the full length scorpine-PhoA fusion band was quantified with total protein normalization as a correction for lane to lane variation. Statistical significance was determined using one way ANOVA with Dunnett's correction where significance is represented by $*P < 0.05$, $**P < 0.01$, and $***P < 0.001$ with experimental replicates. Only significant comparisons are shown. Siders=scorpine antiplasmodial strain using the previously identified siderophore receptor signal. Similar analyses to this figure without using a correction for variations in total protein in each lane and quantifying all three reactive bands in each lane are shown in the manuscript Figure 5B and Supplemental Figures S4 and S5.

Table S1: Oligonucleotides and synthetic dsDNA fragments used in this study. Restriction sites are underlined.

**Primers Used**	**Nucleotide Sequence**<br><br>**5' to 3'**	**Purpose**
PnptII seq F	GAA TTC GGC TTA TTC CCT AAC	PCR and sequence verification of constructs
PhoA Int Seq R2	TTC GGC ATA ATT ACG TGC GG	PCR and sequence verification of constructs
3X GGGGS linker top strand	TCCTGCAGGGGTGGTG GCGGTTCCGGCGGCGG TGGCAGCGGCGGTGGC GGTTCGCCCTGCAGG	Combined with 3X GGGGS linker bottom strand for directional cloning of flexible linker into effector constructs
3X GGGGS linker bottom strand	CCTGCAGGGCGAACCG CCACCGCCGCTGCCAC CGCCGCCGGAACCGCC ACCACCCCTGCAGGA	Combined with 3X GGGGS linker top strand for directional cloning of flexible linker into effector constructs
**gBlocks™ Gene Fragments Used**	**Nucleotide Sequence**<br><br>**5' to 3'**	**Purpose**
Dipeptidyl Carboxypeptidase II	TTTGAACCATATGCCCG TTTTCAGACACACTGCG GCTCTGGCAGCTGTTGC CCTCCCCCTTCTGTCTT CCACGGCGCTGGCTGC CCCTGCCAACCCGCTGT TTCAGGCAAGCCCGCT GCCCTATCAGGCACCC CCCTTCGACAAGATCA AGGACAGTGATTTAAT TAAGACTTC	Insertion of predicted signal sequence into pNB92 vector.
Gamma-Glutamyltranspeptidase	TTTGAACCATATGAAG CGTGTCTGCACCCGGG TTGCGGCCCTGCCGCTC CTCCTCGCCAGCATTTC TGTCTCGAGTCTCGCCC	Insertion of predicted signal sequence into pNB92 vector.

	AGGCTGCGCCAGCGGCCATGCCCTATGACCCGCTGGCCTTCGGCACGGTCAACTTAATTAAGACTTC	
Hypothetical Protein 1	TTTGAACCATATGCTCGTTTTCAATCGTCGTCTTCTTACCGGTGTTGCCGCTCTTGCCATGACGCTGAGCGCCCCTGCTTTCGCTCAGACCACGCAGGAAGCAGCCCCGGCAGCCCCTGCTGCTGCAGCTCCCGCTGCCCCGGCAGCCGCGCCTTTAATTAAGACTTC	Insertion of predicted signal sequence into pNB92 vector.
Hypothetical Protein 2	TTTGAACCATATGCGCTCATTTACCCGCCGTGCCCTTGCTGCTTCGACCTTTGCCGCTGCGGCTCTGGCCGTCATGCCGGTAGCGCATGCAGCCCCCGATGCACTGGGCACCCCCGTTGGCCACATCACCCTGATGGCGAAATCCGCCGATGTGGGTTTAATTAAGACTTC	Insertion of predicted signal sequence into pNB92 vector.
TonB Dependent Receptor Protein 1	TTTGAACCATATGAAGCTTCGCAAGCAGCGCCATGCCCTTACGCTGTCTCTCTTCTGCTCTCCCCTCGCCCTGCTGAGCGCCACCGCTCACGCCGCAGATGCGAAGCTTCCCGTTCATCACAATACGGCACCCGTTTCGCAGCTGCATGCCCAGAACGCTTTAATTAAGACTTC	Insertion of predicted signal sequence into pNB92 vector.
TonB Dependent Receptor Protein 2	TTTGAACCATATGGCCATTTCCCGCATACGCATCTGCCGCGCCTCCATGCTGCTCGGTGCAACGAT	Insertion of predicted signal sequence into pNB92 vector.

	CCTCGCCGGTGCAAGC<br>TTCACGGCTCAGGCCG<br>CCCCCACTACCACGGC<br>CCAGCACAAGGCAAGC<br>CACAAGAGCAGCACAA<br>AGCGCGCCGTACGTTT<br>AATTAAGACTTC	
Hypothetical Protein 3	TTTGAACCATATGTTCA<br>AGACACGTTTTCTGCCC<br>GCACTGGCCCTGACAG<br>CTCTTGTTGCAGCGCCT<br>GTCGCTCATGCGCAGA<br>CTTCCGGATGCAATAA<br>TGCAACGGGAAACGAC<br>ACCATGATCGGCCGTC<br>TGGCCAACAAGGAAAA<br>CTGCATGAATGAGTTA<br>ATTAAGACTTC	Insertion of predicted signal sequence into pNB92 vector.
Ammonia Channel Protein	TTTGAACCATATGAGC<br>CGCGTTTTCAAGGCCCT<br>TGCTCCTCTCGCTCTTT<br>GCGCGCTTCCCCTGCCT<br>GCCTTCGCAGCGCCTG<br>CCGCGATTGACACGGG<br>TGACACGGCCTGGATG<br>CTGGTCAGCACCGCTCT<br>TGTGCTGCTGATGACC<br>ATTCCGGGTGTCTTAAT<br>TAAGACTTC	Insertion of predicted signal sequence into pNB92 vector.
Peptidyl-Prolyl Isomerase	TTTGAACCATATGCGG<br>CTTACCCGCCCGGCCCT<br>TTTCTGCGCCGCTCTCA<br>TCTCCGGCACGACTCTG<br>GCGGCTCCGGCTTTCGC<br>AGCCCCTGCCCCTGCC<br>GCCCCGGCGAATGCGG<br>CTCCGGCCGCTGCCCC<br>GGCTGCCCCTGCCGAT<br>GCCAACCCGCTGTTAA<br>TTAAGACTTC	Insertion of predicted signal sequence into pNB92 vector.

Hypothetical Protein 4	TTTGAACCATATGGTTTCTTTTCCCTGCCGACTCGTCGCCTCGACGGTACTTCTGGCATCGGGCCTGAGCGCCCTGCCTGCCATGGCCGCATCGAAGCAGCAGGCGCTGGTCGATCGGGCAACGTTGGCTGTTCAGGACATTTTTCAGGGGACTAACCCCTTAATTAAGACTTC	Insertion of predicted signal sequence into pNB92 vector.
Hypothetical Protein 5	TTTGAACCATATGAAGAAAGCTCTGCCTGTTCTAGCCGCCCTCATGTTGGGTCTGCCCCTCTCGGCGCAGGCTGCGCCCAAGCCCAAGAACGATCCTGCCTCCGTGCTCAACCGGATTCTGGCCGTTCTCCAGACCGACCCGGACTCGGCCAGCGACAGCTTAATTAAGACTTC	Insertion of predicted signal sequence into pNB92 vector.
Alginate Lyase	TTTGAACCATATGAAGAAAGGCTTGTTCCTTTTTCCGGCCTGCGCCCTTGCCTCCTTTGTGGCGGCAGCCCATGCGGCGCCCGTTTTTGATCCGACCGGCTTTGACCTGCGTGAGACAAAGGCCTTCAGCATGTCGGTCCTGCCCGCCACTGCATTAGGGTTAATTAAGACTTC	Insertion of predicted signal sequence into pNB92 vector.
Hypothetical Protein 6	TTTGAACCATATGACGCGCTTTCTCTCCCTTCTGCTTGTGCTGGGGGCATTGCCTGTTGCTGCTTTTGCGCAGTCTTCGGCGCCGCAATTCGTGCCGCCAAACGGCCTGCCGATGGATACCCCACTTGGCACGGCGCGCAATGCCG	Insertion of predicted signal sequence into pNB92 vector.

	ACGGGTCGGGCGTCTTAATTAAGACTTC	
TonB Dependent Receptor Protein 3	TTTGAACCATATGAAACTCAGACATCGTCTCATTATCTCGGCCAGCCTTCCGGCCCTGCTTATCGCAGCGCATTCTGCGGCGATGGCCCAGAATGTTACGGTTGCACAGCGTGACGCGGCACAGCCTGAGCCGAAAATAACGCGAAAGCTTCAATCCGCCTTAATTAAGACTTC	Insertion of predicted signal sequence into pNB92 vector.
Hypothetical Protein 8	TTAACTTTATAAGGAGGAAAAACATATGCGCCGCATCCTCTTTGCCTTTGCGCTTCTCGCGCTTGCCTCGACCGGGTCGAGCCTCGCTGCTGCCGTGCCGCCCCATTTCGGATCAGCGCTCTCGCAACAGATTTCAAGCGACGGCCCCCGTGCCGTGGCATGGTCGCTTACGCTCTTAATTAAAACCTGCAGGATGCCTG	Gibson assembly of predicted signal sequence into pNB92 vector.
Peroxiredoxin	GATTAACTTTATAAGGAGGAAAAACATATGAAACGCTGCTTTATCCCCGTTTCCGCCGCTCTCCTGTCACTCTCGTTAGCAGCGGCGGTCCCCTCGGCACACGCTGCGCTTAAGCAAGGGGGTGAAGCACCGGATTTCACGCTTCCCGCCACGCAGAATGGGCAGGAAACCAGCTTTTTAATTAAAACCTGCAGGATGCCTG	Gibson assembly of predicted signal sequence into pNB92 vector.
Hypothetical Protein 9	TTAACTTTATAAGGAGGAAAAACATATGCAGGTTCAATCGTTTCGGCGCTGGAGAGGTGCTGCGC	Gibson assembly of predicted signal sequence into pNB92 vector.

	TTGCCGCTCTGGCTTTC GCTGTGGCTCCCGCCAT TGCGCAGGCAGCAGAC GGCCAGTGTGATAACG CGAAATTCCAAACAGA TCAGTCTGGTTTCTGC AAACAGGTCCGTTAAT TAAAACCTGCAGGATG CCTG	
Pentapeptide MXKDX Repeat Protein	TTAACTTTATAAGGAG GAAAAACATATGTCTT TCCGAACACTCGCACTT GCCTGTGCCTTTTCTTC GGCAATGCTGCTGGGA GCCCCTGCCTTCGCTCA GGACACCATGCCACAA GGGACGATGTCGCAGG GCTCAATGGCACATGA CAACATGTCCCACGGC GACATGTCCTCATTAAT TAAAACCTGCAGGATG CCTG	Gibson assembly of predicted signal sequence into pNB92 vector.
Phosphate ABC Transporter Substrate-Binding Protein	TTAACTTTATAAGGAG GAAAAACATATGCAGC TTTCCCGAATTTTCGGC GCCCTGTCGCTCTCTCT GGCCCTGTCATGCGGC GTGTCGCACGCTGCCA CCATCACCGGCGCCGG ATCGAGCTTCGCGGCT CCGATCTATGGCGCTTG GGGTGAGACTGCACAA AAGGCGATCGGCTTAA TTAAAACCTGCAGGAT GCCTG	Gibson assembly of predicted signal sequence into pNB92 vector.
Copper Resistance Protein CopB	GATTAACTTTATAAGG AGGAAAAACATATGAA CATGCGCAGACACACC ATCAACTTTCTGGCTGG CCTTGCCGGCACCCTTT TGCTCTCATCGGGCAG CCATGCGGCGCCTACG GCGCAGGCAAAGTCGA	Gibson assembly of predicted signal sequence into pNB92 vector.

	GTGCAACGATGCCCAG CTATATCGACGGTGTC ATGCCTGTCATGGATTT AATTAAAACCTGCAGG ATGCCTG	

TABLE S2: List of the 20 most-significant predicted extra-cytoplasmic proteins from *Asaia* sp. SF2.1 genome. The predicted, cleaved signal is underlined and in bold. Reproduced from Bongio, 2015.

Gene Name	Locus in Accession Number AYXS00000000.1	Score	Amino Acid Sequence
Dipeptidyl carboxypeptidase II	P792_14605	0.936	**MPVFRHTAALAAVALPLLSSTALA**APANPLFQASPLPYQAPPFDKIKDSD
Gamma-glutamyltranspeptidase	P792_02710	0.929	**MKRVCTRVAALPLLLASISVSSLAQA**APAAMPYDPLAFGTVN
Hypothetical protein 1	P792_12045	0.928	**MLVFNRRLLTGVAALAMTLSAPAFA**QTTQEAAPAAPAAAAPAAPAAAP
Hypothetical protein 2	P792_04190	0.924	**MRSFTRRALAASTFAAAALAVMPVAHA**APDALGTPVGHITLMAKSADVG
TonB dependent receptor protein 1	P792_16355	0.919	**MKLRKQRHALTLSLFCSPLALLSATAHA**ADAKLPVHHNTAPVSQLHAQNA
TonB dependent receptor protein 2	P792_01080	0.913	**MAISRIRICRASMLLGATILAGASFTAQA**APTTTAQHKASHKSSTKRAVR
Hypothetical protein 3	P792_09545	0.907	**MFKTRFLPALALTALVAAPVAHA**QTSGCNNATGNDTMIGRLANKENCMNE
Ammonium channel/transporter	P792_03805	0.905	**MSRVFKALAPLALCALPLPAFA**APAAIDTGDTAWMLVSTALVLLMTIPGV

Peptidyl-prolyl cis-trans isomerase	P792_08510	0.904	**MRLTRPALFCAALISGTTLAAPAFA**APAPAAPANAAPAAAPAAPADANPL
Hypothetical protein 4	P792_11890	0.902	**MVSFPCRLVASTVLLASGLSALPAMA**ASKQQALVDRATLAVQDIFQGTNP
Hypothetical protein 5	ASAP_RS12715<br><br>(From Accession NZ_CBLX010000023)	0.901	**MKKALPVLAALMLGLPLSAQA**APKPKNDPASVLNRILAVLQTDPDSASDS
Alginate lyase	P792_12840	0.899	**MKKGLFLFPACALASFVAAAHA**APVFDPTGFDLRETKAFSMSVLPATALG
Hypothetical protein 6	P792_00230	0.898	**MTRFLSLLLVLGALPVAAFA**QSSAPQFVPPNGLPMDTPLGTARNADGSGV
TonB dependent receptor protein 3	ASAP_0033<br><br>(From Accession NZ_CBLX010000023)	0.893	**MKLRHRLIISASLPALLIAAHSAAMA**QNVTVAQRDAAQPEPKITRKLQSA
Hypothetical protein 8	P792_16160	0.888	**MRRILFAFALLALASTGSSLA**AAVPPHFGSALSQQISSDGPRAVAWSLTLL
Peroxiredoxin (BCP)	P792_17045	0.886	**MKRCFIPVSAALLSLSLAAAVPSAHA**ALKQGGEAPDFTLPATQNGQETSF
Hypothetical protein 9	P792_02680	0.884	**MQVQSFRRWRGAALAALAFAVAPAIAQA**ADGQCDNAKFQTDQSGFLQTGP
Pentapeptide MXKDX repeat protein	P792_05095	0.884	**MSFRTLALACAFSSAMLLGAPAFA**QDTMPQGTMSQGSMAHDNMSHGDMSS

Phosphate ABC transporter substrate-binding protein	P792_05400	0.883	**MQLSRIFGALSLSLALSCGVSHAA**TITGAGSSFAAPIYGAWGETAQKAIG
Copper resistance protein CopB	P792_05375	0.882	**MNMRRHTINFLAGLAGTLLLSSGSHA**APTAQAKSSATMPSYIDGVMPVMD

TABLE S3: Oocyst Counts from three trials of anti-*Plasmodium in vivo* testing.

SF2.1	SF2.1	Siders	Siders	TonBs	TonBs	Hyp1s	Hyp1s	Hyp4s	Hyp4s
33	7	4	19	13	3	2	4	11	1
50	28	19	15	2	0	5	7	0	12
28	8	3	4	16	0	3	14	1	6
17	14	0	5	8	4	0	2	0	10
60	12	7	9	5	8	10	1	3	0
9	12	14	15	0	17	6	0	3	0
33	14	3	14	1	26	0	16	0	0
23	22	7	0	0	9	7	5	2	7
28	11	0	10	7	0	3	2	8	0
16	30	5	20	6	0	4	10	0	5
19	18	12	25	12	6	6	0	2	3
11	15	0	13	4	16	4	8	0	7
2	19	23	15	6	6	0	0	2	9
5	20	11	12	0	20	10	2	3	1
31	7	12	15	3	12	2	1	3	6
28	19	9	4	3	17	0	11	0	6
14	31	3	33	2	8	6	8	0	4
6	19	8	16	8	28	0	3	1	26

3	8	2	13	2	11	16	2	3	4
2	18	0	9	5	20	0	3	5	3
3	17	0		7	18	1		1	3
19	7	5		0		0		0	0
4	28	5		1		6		0	
38	18	0		9		0		1	
23		3		14		12		5	
